# Viruses contribute to microbial diversification in the rumen ecosystem and are associated with certain animal production traits

**DOI:** 10.1186/s40168-024-01791-3

**Published:** 2024-05-09

**Authors:** Ming Yan, Zhongtang Yu

**Affiliations:** 1 Department of Animal Sciences, The Ohio State University, Columbus, OH 43210 USA; 2 Center of Microbiome Science, The Ohio State University, Columbus, OH USA

**Keywords:** Microbiome, Virome, Rumen, Strain diversity, Network analysis

## Abstract

**Background:**

The rumen microbiome enables ruminants to digest otherwise indigestible feedstuffs, thereby facilitating the production of high-quality protein, albeit with suboptimal efficiency and producing methane. Despite extensive research delineating associations between the rumen microbiome and ruminant production traits, the functional roles of the pervasive and diverse rumen virome remain to be determined.

**Results:**

Leveraging a recent comprehensive rumen virome database, this study analyzes virus-microbe linkages, at both species and strain levels, across 551 rumen metagenomes, elucidating patterns of microbial and viral diversity, co-occurrence, and virus-microbe interactions. Additionally, this study assesses the potential role of rumen viruses in microbial diversification by analyzing prophages found in rumen metagenome-assembled genomes. Employing CRISPR–Cas spacer-based matching and virus-microbe co-occurrence network analysis, this study suggests that the viruses in the rumen may regulate microbes at strain and community levels through both antagonistic and mutualistic interactions. Moreover, this study establishes that the rumen virome demonstrates responsiveness to dietary shifts and associations with key animal production traits, including feed efficiency, lactation performance, weight gain, and methane emissions.

**Conclusions:**

These findings provide a substantive framework for further investigations to unravel the functional roles of the virome in the rumen in shaping the microbiome and influencing overall animal production performance.

Video Abstract

**Supplementary Information:**

The online version contains supplementary material available at 10.1186/s40168-024-01791-3.

## Introduction

The rumen microbiome plays an essential role in providing nutrients to ruminants by digesting fibrous feedstuffs that would otherwise remain indigestible and converting low-quality dietary nitrogen into high-quality microbial protein. Nonetheless, the microbial processes involved are inefficient from a ruminant nutritional perspective and contribute to the emissions of methane (CH_4_) and waste nitrogen as urea and ammonia [1]. Extensive research has sought to elucidate the interactions within the rumen microbiome, focusing on its association with diet, animal genetics, and animal phenotype (as reviewed in [2]). Remarkably, except for a very few, all these studies have emphasized the roles of bacteria, archaea, protozoa, and fungi, leaving rumen viruses largely overlooked. As a result, there is a lack of understanding about the ecological and nutritional roles and significance of rumen viruses [2], despite being numerically abundant in the rumen ecosystem [3] and acting as potential apex hierarchy regulators of the rumen microbiome and nutrient recycling.

Microbial viruses significantly impact microbiomes across diverse ecosystems. In ocean settings, viruses lyse approximately 20% of the microbes daily [4], profoundly influencing biogeochemical cycles through the enhancement of carbon and nitrogen recycling via “viral shunt” [5], a process that is modulated by virome diversity in a spatiotemporal manner [6, 7]. In contrast, the human gut virome remains relatively stable [8] but displays considerable variation across an individual’s lifespan [9] and is linked to chronic diseases [10]. Rumen viruses, both abundant [3] and diverse [11], infect diverse rumen microbes including the core rumen microbiome. By lysing rumen microbes at different trophic levels, rumen viruses likely regulate the populations and activities of their hosts (the hosts of rumen viruses unless stated otherwise) and thus the recycling of nutrients and microbial protein [12], which serves as the primary metabolizable protein that ruminants utilize. Thus, unraveling the complexities of virus-microbe interactions within the rumen ecosystem is crucial for deciphering the implication of the rumen virome in animal production performance metrics, encompassing aspects such as feed efficiency, lactation performance, and CH_4_ emissions.

Viruses affect the diversity, population dynamics, and metabolic activities of various microbes through several hypothetical modes of virus-microbe interactions, such as “kill the winner”, “piggyback the winner”, and the “arms-races” dynamics [13]. Specifically, by selectively lysing dominant microbial strains, viruses contribute to the maintenance of microbial diversity. They also facilitate host adaptation and diversification by facilitating horizontal gene transfer [14]. Furthermore, viruses drive microbial diversification through adaptive co-evolution [15]. Prophages, whether cryptic or non-cryptic, serve as accessory gene reservoirs that may carry genes enhancing host survival [16]. Moreover, viruses can impact the metabolism of their host directly by providing auxiliary metabolic genes (AMGs), thereby influencing critical ecological processes in both the environment and gastrointestinal ecosystems, including the human gut and the rumen [11, 17].

Previous studies have documented variations in rumen virome in response to dietary shifts [18] and proposed their potential effects on nutritionally important rumen bacteria [3, 19, 20]. However, the rumen virome remains poorly understood in terms of diversity, interactions with their hosts, and its roles in regulating rumen functions and animal production performance. To bridge this knowledge gap, we recently developed a comprehensive rumen virome database (RVD) by employing the latest bioinformatics pipelines for metagenomic virome analysis across nearly 1000 rumen metagenomes [11]. We revealed a vast diversity of viruses infecting various taxa of bacteria, archaea, and protozoa within the rumen, along with a diverse repertoire of AMGs, including those encoding nutritionally essential enzymes such as cellulases. The revelation of these viruses along with the linkages with their hosts implies a substantial influence on the rumen ecosystem. Building on these findings, we posited that the rumen virome interacts intimately with the microbiome across multiple paradigms and connects to important animal production traits including methane emissions. To test this hypothesis, we systematically characterized and analyzed the prophages at the strain level to decipher viral host specificity at both inter- and intra-species levels. Furthermore, we built and compared microbe-virus and microbe-only networks to ascertain the roles of rumen viruses in shaping rumen microbiome structure. Finally, we analyzed 311 rumen metagenomes reported in nine independent studies, uncovering associations between the rumen virome, microbiome, and critical animal production traits, including feed efficiency, lactation performance, and CH_4_ emissions. Collectively, the results demonstrate that the rumen virome plays pivotal roles in regulating microbial assembly, diversification, and functions, and it is intricately connected with diet and several important animal production traits.

## Method

### Developing and benchmarking custom kraken2 classifiers tailored for the rumen microbiome

While RVD was recently developed from 975 rumen metagenomes, the microbe-virus interaction remains underexplored. To further characterize the species-level microbial profiles in the samples, we developed three custom Kraken2 classifiers based on the Genome Taxonomy Database (GTDB) taxonomy and utilized three databases: the representative genomes of GTDB R207 (https://data.gtdb.ecogenomic.org/releases/release207/, 65,703 genomes), GTDB R207 plus 3588 high-quality rumen metagenome-assembled genomes (MAGs, >90 complete, <5% contamination), and GTDB R207 plus 7176 high-quality dereplicated MAGs assembled from rumen metagenomes in the present study (see supplementary information for details). The latter two databases differ in the number of rumen MAGs (3588 vs. 7176). This allowed us to determine to what extent the increased rumen MAGs would affect the performance of the Kranken2 classifiers. We benchmarked these new Kraken2 classifiers against the standard Kraken2 classifier using the rumen metagenomic data reported in a previous study [21], which were not used in assembling the rumen MAGs or other analyses in the present study. The newly developed Kraken2 classifier that incorporated GTDB R207 and the 7176 rumen MAGs, henceforth referred to as the Rumen Kraken2 Classifier, was used in further analysis.

### Species-level profiling and identification of “core” species of the rumen microbiome

We performed species-level profiling of the 975 rumen metagenomes (collected from 13 ruminant species or animal husbandry regimes across 5 continents) described in the previous study [11] using the Rumen Kraken2 Classifier. The number of sequence reads assigned to individual species was computed using Bracken [22], with the outputs then being compiled and imported to R 4.0.2 [23]. Only the species each represented by >0.001% of the total assigned reads were considered present in a sample. The prevalence of each species and genus was calculated across all metagenomes, and the species and genera with a 100% prevalence were regarded as core/ubiquitous. The relative abundance of each species was calculated as the proportion of the reads assigned to that species relative to the sum of all taxonomically assigned reads. To investigate the influence of sample size on the identification of core species/genera, we employed a custom Python script (see the “Availability of data and materials” section). Briefly, starting with 100 randomly selected samples, we incrementally added 5 random samples each iteration and re-calculated the counts of core species/genera, until all the 975 samples were included. This process was repeated 100 times, and the resulting counts of core species/genus across the iterations were plotted and visualized in R (Supplementary Fig. 1).

### Virome profiling and ecological analysis for alpha- and beta-diversity, differential abundance, and virus-to-host ratio

Of the original studies reporting the 975 metagenomes, nine reported comprehensive metadata, including details of experimental design, dietary treatments, and animal production metrics (Supplementary Table 1). We profiled the viromes within the bulk metagenomes (without enrichment for virus-like particles) derived from these nine studies by mapping the quality-filtered reads to the RVD using CoverM (option: --min-read-percent-identity 0.95, --min-read-aligned-percent 0.75, --min-covered-fraction 0.7; https://github.com/wwood/CoverM) and the trimmed mean method with the minimap2 aligner [24] implemented. The number of reads mapped to the RVD per Gb of metagenomic reads was used as a proxy for viral richness. The Kruskal-Wallis test in R was used to assess the statistical difference in viral richness among treatments or animal groups. We also calculated the corresponding microbial richness based on the microbes that were classified in each metagenome using the Rumen Kraken2 Classifier. Spearman correlation coefficients computed in R were used to identify correlations between viral and microbial richness.

We conducted beta-diversity analyses of the rumen viromes using PCoA, based on Bray-Curtis dissimilarity, through the vegan package [25] in R. We performed PERMANOVA using the adonis2 function of the same package, with 999 permutations in testing for differences among treatments or animal groups. When comparing the rumen viromes among feed efficiencies in beef cattle, the breed was considered a confounding factor, and PERMANOVA was performed with restricted permutations (“strata = breed”).

Differential abundance analysis of microbial and viral profiles across treatments or animal groups was conducted using LinDA [26]. To exclude potentially spurious minor species, only those with a relative abundance exceeding 0.01% in at least 60% of the samples were included. Furthermore, any vOTUs that were found in less than 50% of the metagenomes were excluded. The resulting *p*-values were adjusted for multiple testing with the Benjamini–Hochberg procedure, and significance was declared at an adjusted *p*-value (*q*) < 0.1.

To assess whether variations in diet or animal production performance are associated with changes in the prophage lifecycle, we computed the virus-to-host ratio (VHR), which is defined as the ratio of the prophage genomes coverage rate (determined by CoverM) to the number of reads assigned to their predicted host species (based on the Bracken result). We then compared the VHR across treatments or animal groups in nine studies, focusing on the virus-host linkages in treatments or animal groups that were each represented by at least six rumen metagenomes. We used the Kruskal-Wallis test to assess significance with the *p-*values adjusted for multiple testing using the Benjamini–Hochberg procedure in R.

### Identification, taxonomic classification, and host prediction of prophages identified in the rumen MAGs

Using VirSorter2 V2.2.3 [27], we identified the viral sequences from the 7176 rumen MAGs used to develop the Rumen Kraken2 Classifier, the RUG2 catalog of 1726 rumen MAGs (referred to as RUG2 MAGs hereafter) derived from the 240 samples (referred to as RUG2 samples) [28], and the Hungate1000 genome collection [29], as described in the previous study [11]. The quality of these viral sequences was verified using CheckV 0.8.1 [30], and only those meeting the VirSorter2 category 1 and 2 criteria were retained. These viral sequences underwent further confirmation and validation using VIBRANT [31] V1.2.1 (-virome), and only those confirmed again to be viral were retained as bona fide viral sequences. The confirmed viral sequences were further annotated using DRAM-v V1.2.4 [32]. Two categories of viral sequences were identified as prophages: those flagged as integrated prophages (extracted from the host contigs) by CheckV, and those present in contigs that contain any of the prophage-related genes including those encoding integrase (VOG00021), excisionase (VOG00006, VOG05065), Cro repressor (VOG00002), and Cl repressor (VOG00692), as identified based on the VOGDB database (https://vogdb.org/). We then clustered the identified prophage sequences into vOTU at 95% average nucleotide identity (ANI) across at least 85% of the shortest contigs using scripts from CheckV [30] (https://bitbucket.org/berkeleylab/checkv/src/master/scripts/). The prophage vOTUs were taxonomically classified using PhaGCN2.1 [33] with both the latest International Committee on Taxonomy of Viruses (ICTV) 2022 taxonomy and the old morphology-based ICTV taxonomy, with their host linkages inferred from the MAGs they were identified. A phylogenetic tree that includes the identified host species of the “core” genera was generated using GTDB-tk (option: -classify_wf) [34] and visualized using iTOL [35]. Active prophages, which display a significantly higher coverage than the flanking host genome regions (based on the reads mapping results), were identified with PropagAtE V1.1.0 [36]. We then mapped the reads of the 975 metagenomes to the prophages that have an identified host. A prophage was assumed non-cryptic if it was predicted to be active by PropagAtE in any metagenomes.

### Identification of ARGs carried by prophages

We identified antimicrobial resistance genes (ARGs) present in the prophage sequences using the stringent criteria established in a previous study [37]. Specifically, we predicted the ORFs of the prophage sequences using Prodigal [38] and then aligned them to the CARD 3.2.6 database. Genes conferring resistance via specific mutations were excluded, as recommended previously [39]. Prophage contigs with a greater than 80% identity with a database sequence and 40% coverage were retained for manual curation, as described previously [11].

### Micro-diversity analysis of prophage-carrying strains

First, we identified the MAGs representing individual microbial strains from the 7176 rumen MAGs used to develop the Rumen Kraken2 Classifier and the 1726 RUG2 MAGs using drep (--S_algorithm fastANI --greedy_secondary_clustering -ms 10000 -pa 0.9 -sa 0.98 -nc 0.30 -cm larger). These representative MAGs were combined to form a “MAG mapping database”. To minimize read mis-mapping, we prioritized the RUG2 MAGs using the “--extra_weight_table” flag. Second, we profiled each RUG2 sample at the strain level by mapping its reads to the MAGs mapping database using InStrain [40]. Third, we calculated the non-synonymous to synonymous substitution (pN/pS) ratio (a measurement of gene micro-diversity) of individual genes within each strain detected in each metagenome (without normalization to the expected pN/pS ratio). A strain was deemed present if at least five reads were mapped to at least 50% of its MAGs, as recommended previously [40]. A strain was considered to carry prophage(s) when the breadth of its scaffolds reached > 99%. We computed the pN/pS ratio for each prophage gene with criteria of >99% breadth and 10× coverage. The prophage genomic structure was visualized with the gggenes package in R.

### Host prediction at the strain level using CRISPR spacer matching

We predicted the CRISPR–Cas arrays across the high-quality RUG2 MAGs [41] using MinCED [42]. The identified spacer sequences, at least 30 bp, were then matched to the vOTUs identified from the RUG2 samples (extracted from the RVD) using BLASTn with a threshold of 100% sequence identity. The presence of MAGs and vOTUs in each RUG2 sample was examined using InStrain. We identified genome-level virus-host linkages requiring co-occurrence of both a MAG and a vOTU that have matching spacer sequences in the same RUG2 samples. We also determined the number of microbial strains that had no spacer match but co-existed with strains of the same species that had a spacer match. Only the linkages of sample ERR3275126 were visualized as a network using Cytoscape [43] for illustration.

### Microbe-only and virus-microbe network analysis

Based on the microbial and viral profiles of the RUG2 samples, we constructed microbial-only and virus-microbe networks. To eliminate minor potentially spurious vOTUs and their hosts, we included only major microbial species with a relative abundance exceeding 0.01% in at least 50% of the samples and vOTUs with a trimmed mean (based on CoverM) value exceeding 1 in at least 50% of the samples. Both networks were constructed using SpiecEasi [44] with the sparse graphical lasso (glasso) setting, as described previously [45]. The networks were visualized in R with the package igraph [46]. We computed the network modularity and assortativity with the “fastgreedy.community()” and “assortativity()” functions of igraph, respectively. We also analyzed the data at a 70% prevalence threshold. The degree centrality of microbial nodes was compared between the microbe-only and virus-microbe networks with one-tailed paired *t*-test in R.

## Results and discussion

### A custom rumen Kraken2 classifier tailored to the rumen microbiome enhances the classification and identification of rumen microbes

A custom Kraken2 classifier that incorporates the NCBI RefSeq complete genomes, the Hungate 1000 collection [29], and rumen MAGs substantially improved the classification rate of rumen metagenomic sequences [28]. However, it failed to classify most of the MAGs to the species level. This limitation arises from its reliance on the NCBI taxonomy, which is inadequate to capture the burgeoning numbers of rumen MAGs and thus constraints polyphyletic groupings [47]. To refine species-level identification of virus-microbe interactions, we developed three custom Kraken2 classifiers based on the GTDB taxonomy and utilized three databases: the representative genomes of GTDB R207 (65,703 genomes), GTDB R207 plus 3588 high-quality rumen MAGs (>90 complete, <5% contamination), and GTDB R207 plus 7176 high-quality rumen MAGs (refer to Methods for details). Compared with the standard Kraken2 classifier (https://genome-idx.s3.amazonaws.com/kraken/k2_standard_20231009.tar.gz), the newly developed Kraken2 classifier using GTDB R207 enhanced the species-level classification rate by approximately 55% (Supplementary Fig. 1a). The Rumen Kraken2 Classifier that incorporates GTDB R207 and the additional 7176 rumen MAGs elevated species-level classification rate by another 3%, reaching a total species-level classification rate exceeding 75%. The Rumen Kraken2 Classifier can thus facilitate accurate analysis of virus-microbe linkages and interactions in the rumen ecosystem.

Numerous studies have identified prevalent rumen microbes at the genus level using 16S rRNA gene sequencing [29, 48, 49]. To explore virus-microbe interactions and assess the effects of viruses on dominant microbial species within the rumen, we reanalyzed the 975 metagenomes analyzed in a previous rumen virome study [11]. We discovered a set of ubiquitous species (100% prevalence, across all the 975 metagenomes), most of which belong to the genus *Prevotella* (Supplementary Fig. 2a). Notably, the combined relative abundance of the *Prevotella* species, reaching 80% in some rumen metagenomes, was up to an order of magnitude higher than that of the next most prevalent genus, *Cryptobacteroides* (Supplementary Fig. 2b). Plotting the numbers of core species and genera against increasing sample size revealed a plateau at the species level but not at the genus level (Supplementary Fig. 1b and c), suggesting that most of the core species have probably been accounted for.

### Prophages are prevalent in the rumen ecosystem and may confer survival advantages to their hosts

**Fig. 1:**
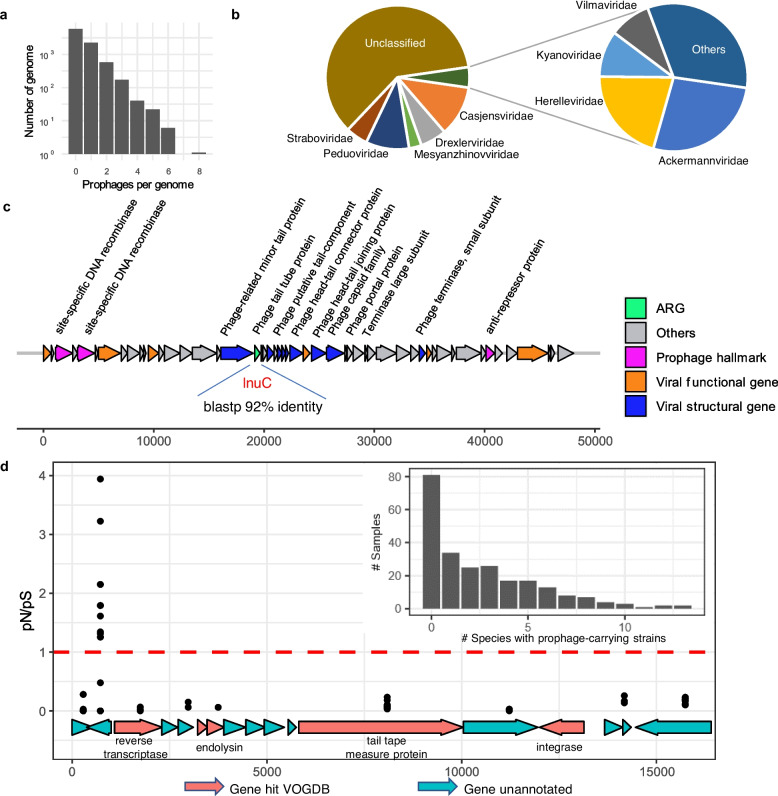
Prophages identified from the rumen microbial genomes. **a**, Number of prophages identified from 8,902 rumen metagenome-assembled genomes (MAGs) including 1,726 RUG2 MAGs [46] and 7,176 MAG assembled in this study (see supplementary information for details). **b**, The taxonomy of the identified prophage vOTUs. **c**, The prophage genome encoding one antimicrobial resistance gene (ARG) identified from an Agathobacter sp900546625 genome. **d**, A prophage gene (second from the 5’ end) under positive selection. This prophage was carried by the genome of one Prevotella sp900317685 strain coexisting with other strains of this species in 46 of the 240 RUG2 samples. Inset figure panel (Upper right corner): distribution of host species that carry various numbers of prophages among the 240 RUG2 samples. See also Supplementary Table 2 for the comparison between the vOTUs taxonomic classification based on the old and new International Committee on Taxonomy of Viruses (ICTV) taxonomy and Supplementary Table 3 for the full list of ARG-carrying prophages and their annotations

The importance of lysogeny and the “piggyback the winner” model have been increasingly recognized in ecosystems densely populated by various microbes [50]. To assess prophage prevalence in the rumen ecosystem, we comprehensively analyzed 8902 rumen microbial genomes and MAGs and found 5185 prophages that represent 4225 vOTUs. Approximately 50% of these genomes and MAGs carry at least one prophage, with one MAG even carrying as many as eight prophages (Fig. 1a). The high prophage prevalence among the rumen microbial genomes/MAGs is comparable with that reported in bacteria in general [51]. All the classifiable prophage vOTUs were classified under the class *Caudoviricetes*, with the majority of prevalent prophage vOTUs classified to the families *Casjensviridae*, *Drexlerviridae*, *Peduoviridae*, *Straboviridae*, while the less prevalent prophage vOTUs classified to other families, including *Mesyanzhinovviridae*, *Ackermannviridae*, and *Herelleviridae* (Fig. 1b). The vOTUs were additionally categorized according to the historical morphology-based ICTV taxonomy, which was utilized during the development of RVD. Changes to the taxonomy can be found in Supplementary Table 2. Of the 5185 identified prophages, 514 were predicted to be active, non-cryptic (based on the significantly higher mapping rates of the prophage genomes than the flanking host genomes), in at least one sample. All 36 ubiquitous bacterial species examined were found to contain prophage sequences, and the majority carry both cryptic and non-cryptic prophages (Supplementary Fig. 3). The propensity for a host genome to carry non-cryptic prophages appeared to vary among bacteria, with the genomes from the phylum *Bacteroidota* more likely carrying non-cryptic prophages than those from *Firmicutes_A*.

In a recent study, we identified ARGs in some of the viral MAGs [11]. The current study specifically focused on the ARGs carried by complete prophages. Our analysis revealed the presence of ARGs in multiple prophage genomes (Supplementary Table 3), including one prophage genome from a MAG of *Agathobacter sp900546625* (Fig. 1c). This particular prophage carries an ARG sharing 92% amino acid identity with LnuC, an ARG that confers resistance to lincomycin through nucleotidylation in *Streptococcus agalactiae* UCN36 [52]. Since this ARG is demarcated by viral hallmark genes on both ends, it is unlikely part of the host genome. While most of the identified prophages were potentially cryptic, they may still confer adaptive advantages to their host, such as by providing ARGs and accessory genes [16, 53, 54]. The diversity and prevalence of these genes, especially ARGs and genes involved in nutrient acquisition, warrant further investigation.

We further assessed the co-existence of multiple strains (individual MAGs) within the 1726 RUG2 MAGs derived from 240 RUG2 samples [28]. We found that most of these samples had multiple species each containing multiple prophage-carrying strains (Fig. 1d). Given that the MAG database only retains a limited subset of species for strain identification, the actual strain-level diversity is likely higher. Nevertheless, we found multiple strains of *Prevotella sp900317685*, including one strain carrying a cryptic prophage, co-existing with other strains in 46 of the RUG2 samples. Examining the pN/pS ratio of the genes of this cryptic prophage, we found one unannotated gene with a pN/pS ratio exceeding one in most of these 46 samples (Fig. 1d), which indicates that this gene is undergoing positive diversifying selection. While the function of this gene is unknown, its presence may hint at survival advantages conferred by this prophage gene. Interestingly, this phage also encodes a reverse transcriptase, which may be a part of diversity-generating retroelements that have been previously shown to promote genetic variation, particularly in the regions involved in host genetic recognition [55]. Besides, temperate phages can also promote horizontal gene transfer (HGT) and microbial diversification not just through specialized and generalized transduction, the latter of which is rare, but also through lateral transduction and conjugative transfer, both of which are common [15, 56]. These processes can obscure the demarcation between host chromosomes and mobile genetic elements [56–58]. Collectively, prophage-mediated HGT and the introduction of new genes during lysogenic conversion contribute to a beneficial relationship at the population level.

### Rumen viruses regulate microbiome at both species and strain levels

**Fig. 2:**
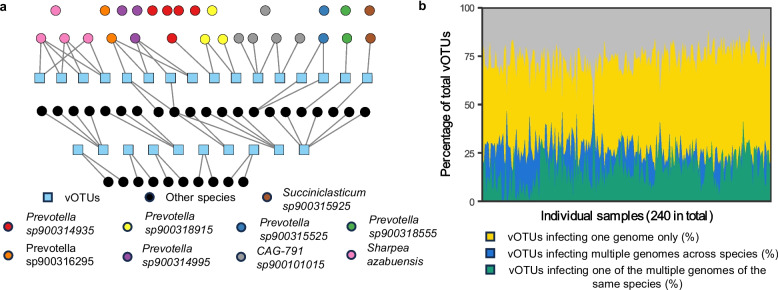
Strain level host specificity of rumen viruses. **a**, Inter- and intra-species host specificity of the rumen viruses exemplified with one of the RUG2 samples [46], ERR3275126. Each circle represents one microbial genome and is color-coded based on species, while each square represents one vOTU. Connected vOTUs and microbial genomes have matches between the vOTU protospacer sequences and the corresponding microbial spacer sequences. Unconnected circles represent coexisting microbial genomes whose spacer sequences did not match any protospacer sequences. **b**, The percentage of vOTUs in each of the RUG2 samples (240 in total) infecting a single genome (or MAG) of bacteria, multiple genomes (or MAGs) of different bacterial species, or one of the multiple genomes (MAGs) of the same species (i.e., co-existing bacterial strains of the same species that lack a spacer that matches a protospacer sequence)

The intricate interplay between microbial defense mechanisms and viral countermeasures contributes to their co-evolution and shapes microbiome structure, especially at the strain level [59, 60]. To explore these co-evolutionary dynamics, we examined the virus-host interactions across 1422 high-quality MAGs and tens of thousands of vOTUs that we identified from the RUG2 samples. Employing CRISPR–Cas spacer matches (requiring 100% sequence identification), we assessed the co-existence and infection patterns between these MAGs and vOTUs at the strain level. We identified viruses with both inter- and intra-species host specificity, as exemplified by the virus-host linkages in one sample (Fig. 2a). Notably, many microbial genomes and MAGs contained CRISPR–Cas spacers that match multiple vOTUs. Because our analysis focused on high-quality MAGs, which are typically derived from highly abundant bacteria, the strain-level virus-host linkages we identified are likely skewed towards those abundant in the rumen ecosystem, such as strains of *Prevotella* species (e.g., *Prevotella sp900314935* and *Prevotella sp900314995*). Some MAGs had no match with the protospacer sequences of vOTUs predicted to infect the corresponding host species, indicating immunity or absence of previous infection. Since CRISPR spacers document past phage infections, concurrent detection of a matching CRISPR spacer and a protospacer in co-existing virus and microbe indicates a long-term coevolutionary relationship [61], which promotes both viral and microbial diversity. Moreover, we found many protospacers with a single mismatch with their corresponding CRISPR–Cas spacer. This could indicate point mutations in the viral genomes to evade the CRISPR–Cas system.

The prevalence of CRISPR–Cas system among the rumen MAGs and genomes, together with previously identified restriction-modification systems (e.g., methyltransferase) in many rumen viral genomes [11], suggests that the “arms-races” model also plays a vital role in the rumen ecosystem. In analyzing the RUG2 samples, we found that about 80% of the vOTUs would infect just one single host strain, represented by one genome or MAG, and thus a single host species (Fig. 2b), while other vOTUs showed both inter- and intra-species host specificity. Some rumen viruses have a broad host range, as documented in previous studies [11, 62]. Their broad host range may be attributable to, among others, mutations and rearrangements of receptor-binding proteins [63] and “sensitivity acquisition,” a process wherein bacteria initially resistant to phage infection become susceptible through receptor exchange with susceptible co-inhabitants [64].

The strain-level microbial diversity in the rumen may be associated with host production traits. For example, no correlation was found between methane emission and microbial abundance at the sub-genus level [65], but subsequent research revealed that such a correlation existed at the strain level [28]. Therefore, by regulating microbiomes at both strain and species levels, rumen phages could also have an intricate relationship with animal production traits. Furthermore, the dynamic equilibrium between microbial defense and viral counter-defense may result in oscillation in clonal abundance as a result of the genetic sweeps [66]. Overall, the complex nested infections (phages infecting multiple strains/species and microbes infected by multiple phages) underscore the intricate virus-microbe interactions, which is further illustrated in the next section, and signify an important role of viruses in promoting trophic cascades as posited in a previous study [67].

### Rumen viruses facilitate microbial interactions, as shown by virus-microbe networks

**Fig. 3:**
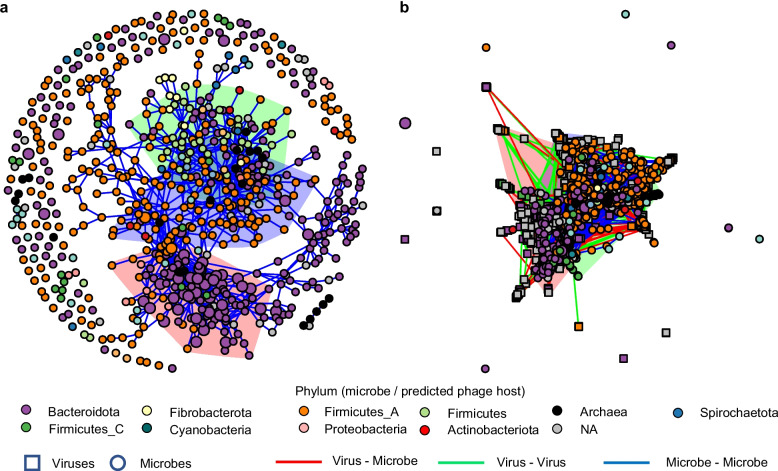
Co-occurrence networks showing the modular organization of rumen microbiome and microbe-virus interactions. **a**, Rumen microbe-only network. **b**, Microbe-virus network. Both networks were built with the microbes and viruses identified with a prevalence greater than 50% in the 240 RUG2 samples [46]. Microbial nodes are denoted as circles, and viral nodes are denoted as squares. The microbial species of the same phylum or predicted hosts of the viruses are displayed with one distinct color. Large circles represent core bacterial species ubiquitous in the 975 metagenomes used in developing the RVD [11], while small circles represent non-core microbial species. The colors of the edges designate different connections between different nodes. The three largest modules in each network are highlighted in red, green, and blue. See also Supplementary Fig. 4 for the largest three modules of the microbe-virus network

To investigate microbial interactions, we constructed a microbial co-occurrence network using the RUG2 samples. This network contains 671 microbial nodes, 119 of which are singletons and not linked to the main network (Fig. 3a). With an average degree centrality of 3.13 (± 3.24) and a modularity index of 0.71, the network displays a robust community structure. The network comprises three large, highly interconnected modules or discrete clusters of nodes. Each module has over 45 nodes, suggesting niche differentiation. We noted a moderate assortment among nodes based on their phyla (assortment coefficient c_a_ = 0.43). The largest module comprises 109 nodes, including primarily species within the genera of *Bacteroidota*, followed by species within genera of *Firmicutes*, *Firmicutes_A*, *Firmicutes_C*, *Fibrobacter*, *Actinobacteriota*, and archaea. The second largest module encompasses 93 nodes, mostly core species of *Prevotella* and *UBA4334* (a genomic genus in the family *Bacteroidaceae* in GTDB). This module also contains several genera of *Firmicutes_C*, *Proteobacteria*, and archaea. The smallest module has 47 modes and features a diverse array of species from multiple phyla, including *Firmicutes*, *Firmicutes_A*, *Firmicutes_C*, *Actinobacteriota*, and *Bacteroidota* and archaea. All three modules contain unclassified species, indicating that some rumen microbes are not represented by the current GTDB database. Although the modules have the same set of phyla, they each have distinct genera, implying niche differentiation at finer taxonomic scales.

We also constructed a virus-microbe cooccurrence network to examine virus-microbe interactions (Fig. 3b). This network includes 570 viral nodes and the 671 microbial nodes of the microbe-only network. In this network, 22 microbial nodes do not connect to other microbial nodes. When considering only the microbial nodes, the average degree centrality is 5.23 (± 3.94), significantly higher than that of the microbe-only network (paired *t*-test, *p* < 0.001). With a modularity index of 0.60, relatively lower compared to that of the microbe-only network, the virus-microbe network still reveals a relatively robust community structure. Unlike in the microbe-only network, the three largest microbial modules in the virus-microbe network have a similar taxonomy composition, and each contains multiple microbe-virus and virus-virus edges (Supplementary Fig. 4). Moreover, the three modules are less separated (assortment coefficient *c*_a_ = 0.34) compared to the microbe-only co-occurrence network. The microbe-virus edges can signify co-existence strategies, either as prophages within host microbes or as lytic viruses alongside virus-resistant microbes. In the latter scenario, it may be because virus-resistant microbes benefit from increased nutrient availability due to decreased competition and nutrients released from the microbes lysed by the lytic viruses, as shown previously [68]. Although viruses may act antagonistically at the cell level, the augmented connectivity and reduced assortativity of the microbial nodes in the virus-microbe network, relative to the microbe-only network, suggest that viruses may facilitate microbial interactions and allow diverse microbes to occupy the same niches. This inference is further supported by the modular and nested virus-microbe infection network as shown in Fig. 2a. Overall, these intricate virus-microbe interactions extend beyond the predator-prey relationship and indicate that rumen viruses and microbes could be mutualistic at the microbiome level, corroborating the previous finding in the human gut ecosystem [56]. Interactions between phages can arise from superinfection immunity induced by prophages or co-infection of the same bacteria species. Repeating the analysis with an increased prevalence threshold from 50 to 70%, we noted increased connectivity and decreased assortativity of the virus-microbe network (Supplementary Fig. 5b), relative to the microbe-only network (Supplementary Fig. 5a). This indicates that the initial prevalence threshold did not bias the results.

Several microbial (Supplementary Fig. 6a) and viral (Supplementary Fig. 6b) nodes exhibit both a high degree of centrality (>15) and a betweenness centrality (>15,000). These nodes include *Prevotella sp902778255*, *Prevotella sp900319305*, *GCA-900199385 sp017512985*, *UBA1711 sp001543385*, *RUG572 sp902802945*, and *Schwartzia succinivorans*. These species could be viewed as “keystone” bacterial species, crucial for maintaining community structure. Some of the nodes contain ubiquitous species but with a lower average degree centrality and betweenness centrality, 10 and 3600, respectively. Modularity analysis suggests that while most of these ubiquitous species occupy distinct and essential niches, they may not be keystone species. Notably, two keystone viral species were predicted to infect *Ruminococcus_E sp900314795* and *CAG-791 sp900101015*. Given the intricate interplay between microbes and viruses, future rumen microbiome research should concurrently analyze both entities to understand the inconsistent and transient effects of microbial interventions reported in a previous study [69]. Moreover, stochastic events affect the early colonization of the rumen and have a lasting influence on the rumen microbiome [70], but viruses were not taken into account. Future studies on the rumen ecosystem development should also include analyses of rumen viruses.

### Dietary composition, animal production performance, and CH4 emissions are linked to the macro- and micro-diversity profiles of the rumen virome

**Fig. 4:**
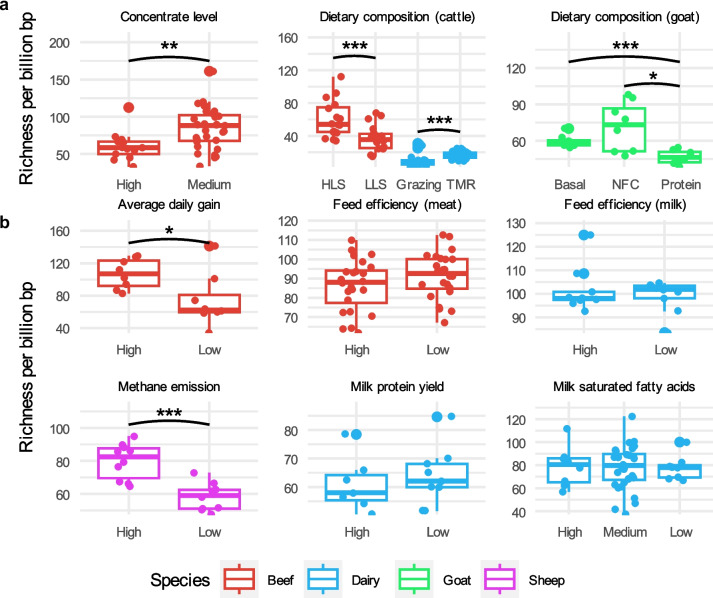
Rumen viral richness is associated with both dietary composition and animal production traits. **a**, The effect of dietary composition on viral richness. **b**, Viral richness variations between animals with differing production traits. Box plots indicate the median (middle line), 25th and 75th percentiles (box), and 5th and 95th percentiles (whiskers) as well as individual observations (dots). Statistical significance was tested using the two-sided non-parametric Wilcoxon signed-rank test. *p* values below 0.05, 0.01, and 0.001 are indicated as “*”, “**”, and “***”, respectively. See also Supplementary Table 1 for detailed information about the studies included. TMR: total mixed ration, primarily consisting of corn silage and concentrate (grain); LLS: low lipid starch diet; HLS: high lipid starch diet; basal: a basal diet with 9.6% crude protein (CP), 14.1% nonfiber carbohydrates (NFC), and 67.3% neutral detergent fiber (NDF); NFC: a diet with 10% CP, 28.3% NFC, and 53.6% NDF; Protein: a diet with 15.6% CP, 16.3% NFC, and 59.3% NDF

The interrelationship between the rumen microbiome, diet, animal production performance, and CH_4_ emissions represents a key focus of rumen microbiome research. However, few studies have examined the connections of the rumen virome with the above factors or production traits. Only one study in the literature has shown that dietary energy levels can affect both the rumen virome and microbiome [18]. In the current study, we analyzed the rumen virome profiles of 311 rumen metagenomes from 9 studies that reported a detailed experimental design to investigate the association between the rumen virome, diet, and animal production traits (Supplementary Table 1). To mitigate variability arising from differences in diet and animal genetics across the studies, we analyzed the data on a study-by-study basis. Overall, dietary composition affected virome richness, but the extent of the effect varied (Fig. 4a). For instance, beef cattle fed high-concentrate diets had a lower virome richness compared to those fed medium-concentrate diets. In dairy cattle, high-lipid and high-starch diets corresponded to increased virome richness, while grazing led to a lower richness compared to total mixed ration (TMR, primarily consisting of corn silage and corn grain). Non-fiber carbohydrate (NFC) levels did not affect rumen virome richness in goats, but the levels of dietary protein and neutral detergent fiber (NDF, representing cellulose, hemicellulose, and lignin of plant fiber) appeared influential. Diets likely affect the rumen virome indirectly by affecting their hosts.

The average daily gain in beef cattle and CH_4_ emissions from sheep correlated positively with rumen viral richness, but feed efficiency in both beef cattle and dairy cows, as well as milk protein yield and saturated fatty acid yield, showed no association with virome richness (Fig. 4b). Animal production performance is affected by diet and a wide range of host factors such as age, metabolism, physiology, and health [71–73]. The lack of significant association between the rumen virome and these animal production performance metrics may be attributable to those animal factors. In examining the correlation between microbial richness and viral richness in the same rumen metagenomes, we found inconsistent results (Supplementary Fig. 7). Specifically, a significant correlation between microbiome and virome richness was observed only in some of the metagenomes, with no consistent directionality, suggesting that other factors likely affect their interactions and population dynamics. Given the highly individualized nature of the rumen virome [11, 56], interactions between rumen viruses and microbes, especially those at low abundance, may be affected by stochasticity constrained by the deterministic effects of diet and animal genetics.

**Fig. 5:**
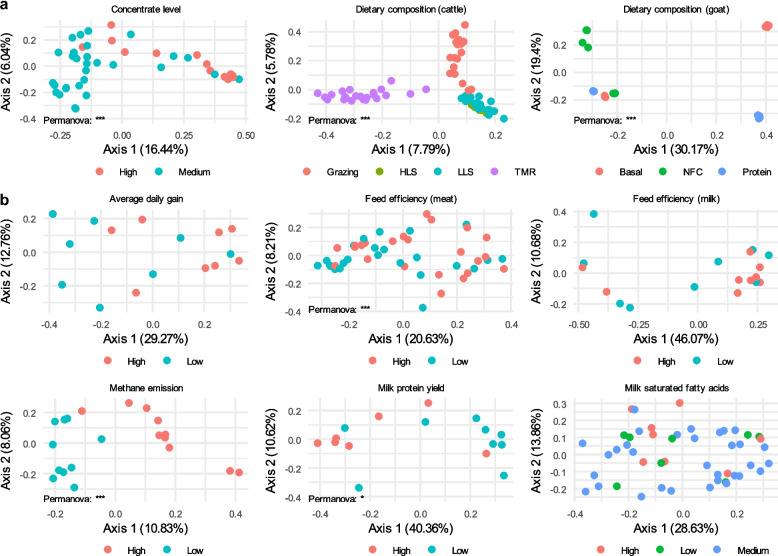
Principal coordinates analysis comparing rumen virome compositions between dietary compositions and between animal production traits. **a**, Comparison of rumen viromes between dietary compositions. **b**, Comparison of rumen viromes between animal production traits. Permutational multivariate analysis of variance (PERMANOVA) was used to compare the overall viromes. *p* values below 0.05, 0.01, and 0.001 are indicated as “*”, “**”, and “***”, respectively. See also Supplementary Table 1 for detailed information about the studies included. TMR: total mixed ration, primarily consisting of corn silage and concentrate (grain); LLS: low lipid starch diet; HLS: high lipid starch diet; basal: a basal diet with 9.6% crude protein (CP), 14.1% non-fiber carbohydrates (NFC), and 67.3% neutral detergent fiber (NDF); NFC: a diet with 10% CP, 28.3% NFC, and 53.6% NDF; Protein: a diet with 15.6% CP, 16.3% NFC, and 59.3% NDF

We further analyzed the beta-diversity of the rumen viromes among animal groups using principal coordinates analysis (PCoA) based on Bray-Curtis dissimilarity. Particularly, rumen virome composition differed between diets (Fig. 5a), feed efficiencies in beef cattle (breeds as a confounding factor), CH_4_ emissions from sheep, and milk protein yields. Conversely, no differences were observed among average daily gains in beef cattle, feed efficiencies, or milk saturated fatty acid yields in lactating dairy cows (Fig. 5b). Although some studies have reported correlations between rumen microbiome composition and the above animal production traits [65, 74–76], other studies have not [77]. The divergence in the association between animal production performance and the rumen virome, relative to the rumen microbiome, may be attributable to the more individualized rumen virome profiles than the microbiome profiles.

### Rumen viruses have different effects on microbial species depending on dietary conditions and animal production performance

**Fig. 6:**
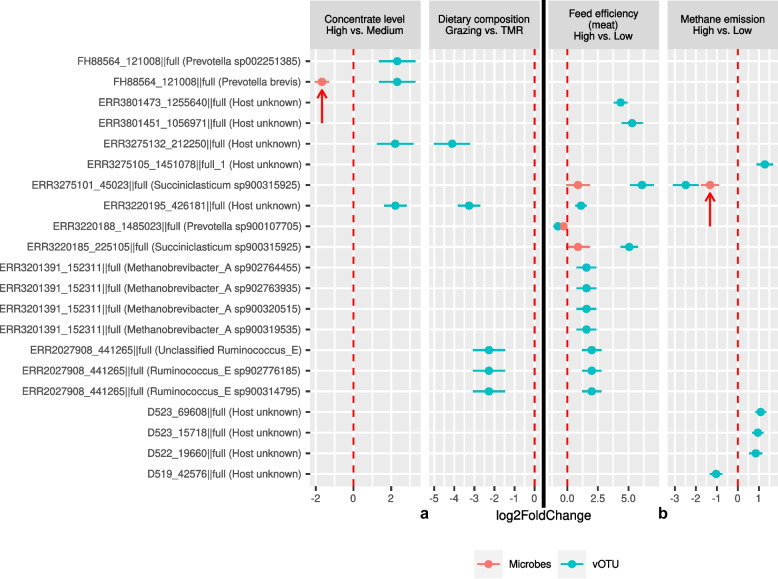
Phages infected bacteria have varying abundant in ruminants fed different diets or with different efficiency. Differential abundance analysis identified several vOTUs (blue) that were differentially abundant in ruminants fed different diets (**a**) and animals differing in feed efficiencies or methane emissions (**b**). The log2-fold changes of their predicted hosts are denoted in red, and those also significantly differentially abundant between animal cohorts are indicated by red arrows. See also Supplementary Table 2 for detailed information about the studies included. TMR: total mixed ration, primarily consisting of corn silage and concentrate (grain)

In addition to modifying microbiome structure, the viruses in the rumen may directly modulate the fermentation therein by affecting the abundance of key microbial species. Using differential abundance analysis, we identified several dozens of vOTUs with different abundance (*q* < 0.1) across varying dietary compositions (Fig. 6a) and animal production metrics (Fig. 6b). Because a considerable proportion of the vOTUs could not be classified at any taxonomy rank above vOTUs, differential abundance was analyzed only at this granularity. The hosts of some differentially abundant vOTUs also displayed varied abundance (*q* < 0.1; indicated by red arrows in Fig. 6). Notably, vOTU FH88564_121008||full, predicted to infect *Prevotella brevis*, was more prevalent in the medium concentrate group, whereas *Prevotella brevis* itself was more prevalent in the high concentrate group. Conversely, vOTU, ERR3275101_45023||full, and its predicted host, *Succiniclasticum sp900315925*, exhibited the same trend: more prevalent in the low CH_4_ emission group than in the high CH_4_ emission group. Since these two vOTUs were predicted to be prophages, their divergent trends may signify disparate life cycles. The hosts of some vOTUs could not be predicted, probably because their abundance was below the 0.01% threshold. None of the AMG-encoding vOTUs was differentially abundant, possibly due to their limited representation in the dataset used in the analysis.

We evaluated potential associations between diet or animal production performance and lifecycle alterations of prophages by comparing the VHR among the predicted virus-host linkages across the 311 rumen metagenomes that were used for diversity analysis. We found a higher VHR (*q <* 0.01) for the prophages predicted to infect *Prevotella sp002251295* and *Prevotella sp900107705* in the animals fed a concentrate-based diet (the NFC diet) compared with those fed a forage-based diet (Supplementary Fig. 8a). This disparity is likely attributable to the increased feed fermentation and production of short-chain fatty acids (SCFAs), which are known to induce prophages [78]. Indeed, certain food and food extracts have been shown to induce prophages in the human microbiome [79]. Dietary fructose and SCFAs also potentiated prophage induction in *Lactobacillus reuteri*, a gut microbe [78]. Additionally, subacute rumen acidosis, generally induced by rapid SCFA production in animals consuming high-concentrate diets, has been shown to substantially increase rumen viral abundance [11]. Thus, although shifts in the rumen virome largely mirror alterations in microbiome structure, changes in the rumen environment may modulate viral lifecycle dynamics, which can in turn affect the rumen microbiome structure. Intriguingly, the VHR between prophage vOTU FH88564_121008||full and its host, *Prevotella brevis*, remained unaffected by the concentrate levels, despite their differential abundance at the two concentrate levels. This can likely be attributed to the concurrent presence of multiple strains of the bacterial host species, and they do not carry the same prophage, as shown in the previous section. Moreover, sheep with varying CH_4_ emissions exhibited significantly different VHR (Supplementary Fig. 8b), which may be ascribed to alterations in viral lifecycle dynamics induced by shifts in microbial metabolisms [65].

The turnover of rumen microbes caused by viral lysis can have a far-reaching effect on certain rumen functions, especially fermentation and microbial protein synthesis. Although marine phages are estimated to lyse approximately 20% of marine bacteria daily [4], the lysis rate of rumen microbes attributable to viruses remains undetermined. Given the high abundance of both viruses and microbes in the rumen, viral lysis therein is likely substantial. Two key questions thus arise: What is the virus-mediated turnover rate of both total and specific rumen microbes, particularly those pertinent to animal production performance and CH_4_ emissions? To what extent do lysogenic and lytic cycles predominate in the rumen ecosystem? Early studies used transmission electron microscopy to count phages [3] or total phage DNA concentration as a proxy of phage population size in the rumen [19]. However, a high phage count does not necessarily correlate with an elevated microbial host mortality rate. For example, it has been shown that less than 1% of the cyanobacterial cells were infected even in the presence of high concentrations of free phage particles [80]. It is worth noting that the above two methods likely underestimate phage abundance because they primarily account for free lytic virions. In contrast, VHR calculated from metagenomic sequences can quantify not only free virions but also temperate phages and intracellular lytic phages [81]. Furthermore, the single-cell polony method can help identify lineage-resolved viral infections across thousands of cells of various microbes simultaneously [80]. Leveraging these methodological advancements, future studies should aim to quantify the virus-host ratio, host mortality rate, and their associations with net microbial protein synthesis in the rumen ecosystem. Such data will provide invaluable insights into the role of viral lysis in intra-ruminal nitrogen cycling across different feeding regimes.

## Conclusions

In conclusion, this study delves into the still largely unknown roles of viruses within the rumen ecosystem. Comprehensive analyses of rumen metagenomes, both viral and microbial sequences, revealed intricate virus-microbe relationships, providing new insights into the diversity, co-occurrence, and interactions between these components. Furthermore, this study shows that rumen viruses may exert regulatory influence on rumen microbes at both strain and community levels through both antagonistic and mutualistic interactions. Notably, the rumen virome displays adaptability in response to dietary changes and exhibits associations with crucial animal production traits, such as feed efficiency, lactation performance, weight gain, and methane emissions. These findings establish a robust foundation for future research endeavors aimed at deciphering the functional roles of the rumen virome in shaping the rumen microbiome and its profound impact on overall animal production performance. Future research should also investigate single-strain DNA or RNA viruses in the rumen as they are currently underrepresented in the RVD and rumen metagenomes.

### Supplementary Information

**Supplementary Material 1.** 

**Supplementary Material 2.** 

**Supplementary Material 3.** 

**Supplementary Material 4.** 

**Supplementary Material 5.** 

**Supplementary Material 6.** 

**Supplementary Material 7.** 

**Supplementary Material 8.** 

**Supplementary Material 9.** 

**Supplementary Material 10.** 

**Supplementary Material 11.** 

**Supplementary Material 12.** 

## Data Availability

The sequencing data used in this study are available on NCBI SRA with accession number PRJNA202380, PRJNA627251, PRJNA597489, PRJNA448333, PRJEB23561, PRJEB21624, PRJEB33080, PRJNA526070, and PRJNA492173. The rumen virome database used in this study is available at https://zenodo.org/records/7412085. All codes used for this study are available online at https://github.com/yan1365/rumen_virome_eco.
