# Supplementary material for: Viruses contribute to microbial diversification in the rumen ecosystem and are associated with certain animal production traits
Source: Microbiome. 2024 May 9;12:82. doi: 10.1186/s40168-024-01791-3 (PMC11080232; doi:10.1186/s40168-024-01791-3)
Supplement: Supplementary file 2 — Supplementary Material 1. [file 40168_2024_1791_MOESM1_ESM.docx]

Supplementary Method

**Build the custom kraken2 and bracken classifiers based on GTDB taxonomy.**

Firstly, we downloaded reference genomes of GTDB-R207 (<https://data.gtdb.ecogenomic.org/releases/release207/>). Then we supplemented the database with MAGs database assembled from rumen. Specifically, the supplemented database consist of the rumen MAGs generated or collected in RVD (1), RUG2 (2) and Hungate collection (3). The quality of the acquired MAGs was assessed with CheckM (4), and only those determined to be of high quality (completeness ≥90% and contamination ≤5%) was retained. The redundant conspecific genomes were removed from the remaining high quality rumen MAGs by removing genomes shared ≥99.9% average nucleotide identity (ANI) with dRep V3.4.0 (5) (options: -pa 0.999 –SkipSecondary). In total, 7176 high quality non-redundant rumen MAGs were retained. The taxonomy was assigned to the 7176 MAGs with GTDB-Tk (6). The rumen MAGs (including MAGs without species or higher level classification) were supplemented with representative genomes of GTDB-R207 to build the custom kraken2 and bracken classifiers. The GTDB taxonomy taxdump files were generated with TaxonKit (7) and modified to accommodate the unclassified rumen microbial genomes using a custom python script.

**References**

1. Yan M, Pratama AA, Somasundaram S, Li Z, Jiang Y, Sullivan MB, et al. Interrogating the viral dark matter of the rumen ecosystem with a global virome database. Nature Communications. 2023;14(1):5254.

2. Stewart RD, Auffret MD, Warr A, Walker AW, Roehe R, Watson M. Compendium of 4,941 rumen metagenome-assembled genomes for rumen microbiome biology and enzyme discovery. Nat Biotechnol. 2019;37(8):953-61.

3. Seshadri R, Leahy SC, Attwood GT, Teh KH, Lambie SC, Cookson AL, et al. Cultivation and sequencing of rumen microbiome members from the Hungate1000 Collection. Nature biotechnology. 2018;36(4):359-67.

4. Parks DH, Imelfort M, Skennerton CT, Hugenholtz P, Tyson GW. CheckM: assessing the quality of microbial genomes recovered from isolates, single cells, and metagenomes. Genome Res. 2015;25(7):1043-55.

5. Olm MR, Brown CT, Brooks B, Banfield JF. dRep: a tool for fast and accurate genomic comparisons that enables improved genome recovery from metagenomes through de-replication. Isme J. 2017;11(12):2864-8.

6. Chaumeil PA, Mussig AJ, Hugenholtz P, Parks DH. GTDB-Tk: a toolkit to classify genomes with the Genome Taxonomy Database. Bioinformatics. 2019.

7. Shen W, Ren H. TaxonKit: A practical and efficient NCBI taxonomy toolkit. Journal of Genetics and Genomics. 2021;48(9):844-50.
