# Supplementary figures and images for: Viruses contribute to microbial diversification in the rumen ecosystem and are associated with certain animal production traits
Source: Microbiome. 2024 May 9;12:82. doi: 10.1186/s40168-024-01791-3 (PMC11080232; doi:10.1186/s40168-024-01791-3)

**a**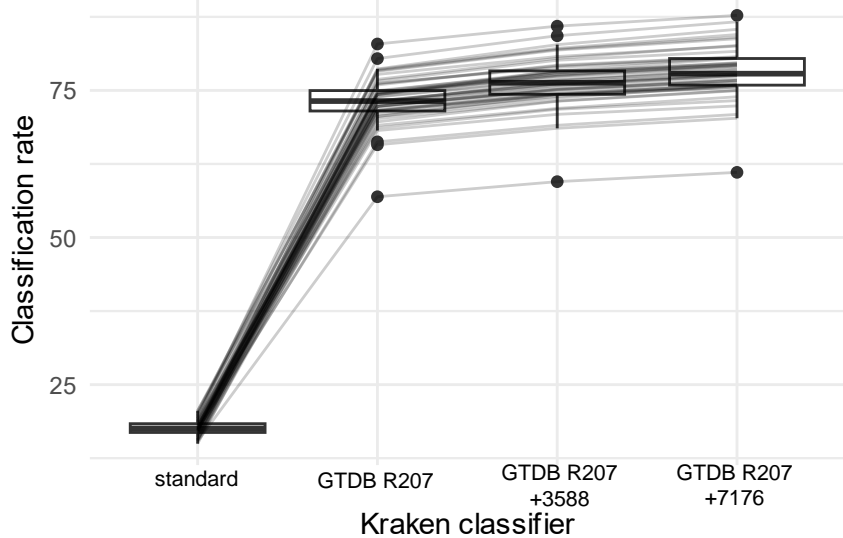**b**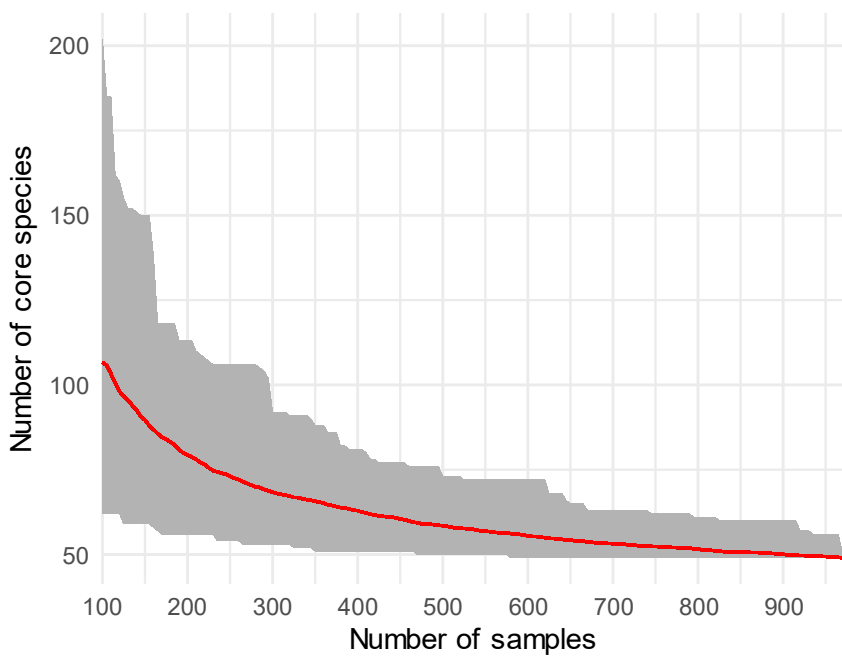**c**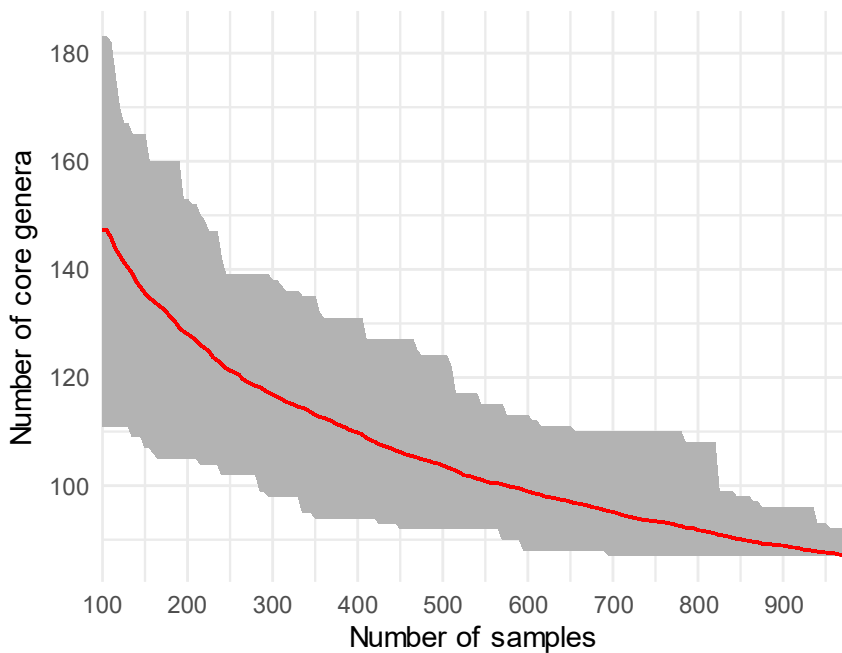

Supplement: Supplementary file 3 — Supplementary Material 2. [file 40168_2024_1791_MOESM2_ESM.pdf]

a

core species

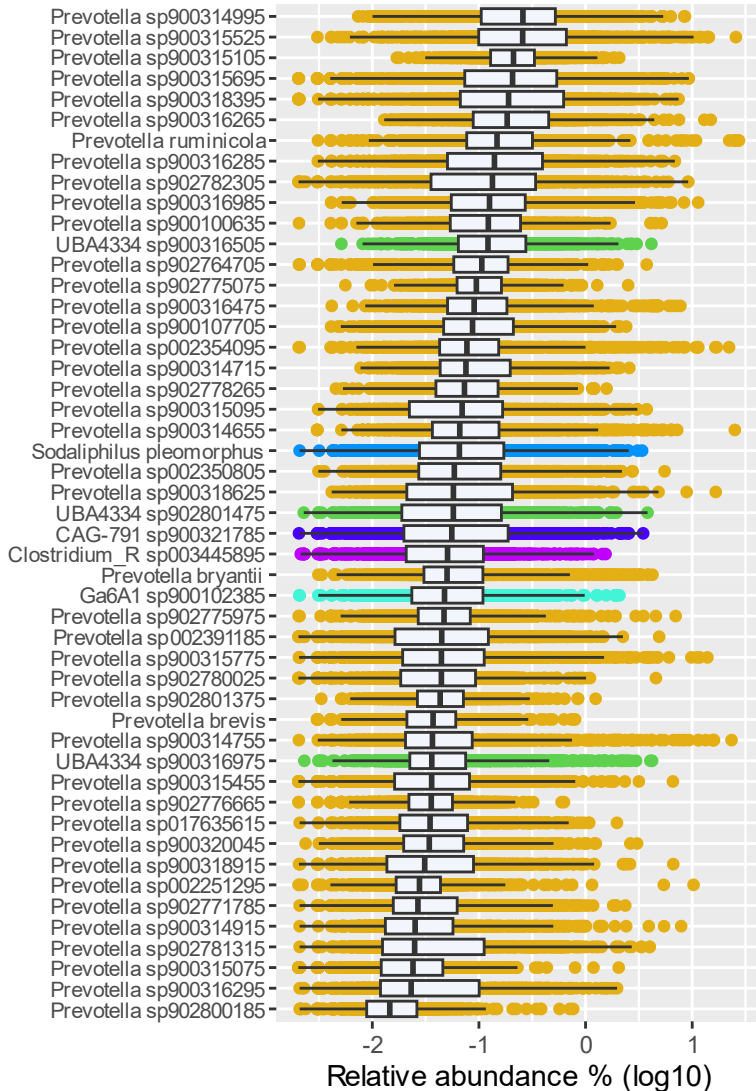

b

core genera

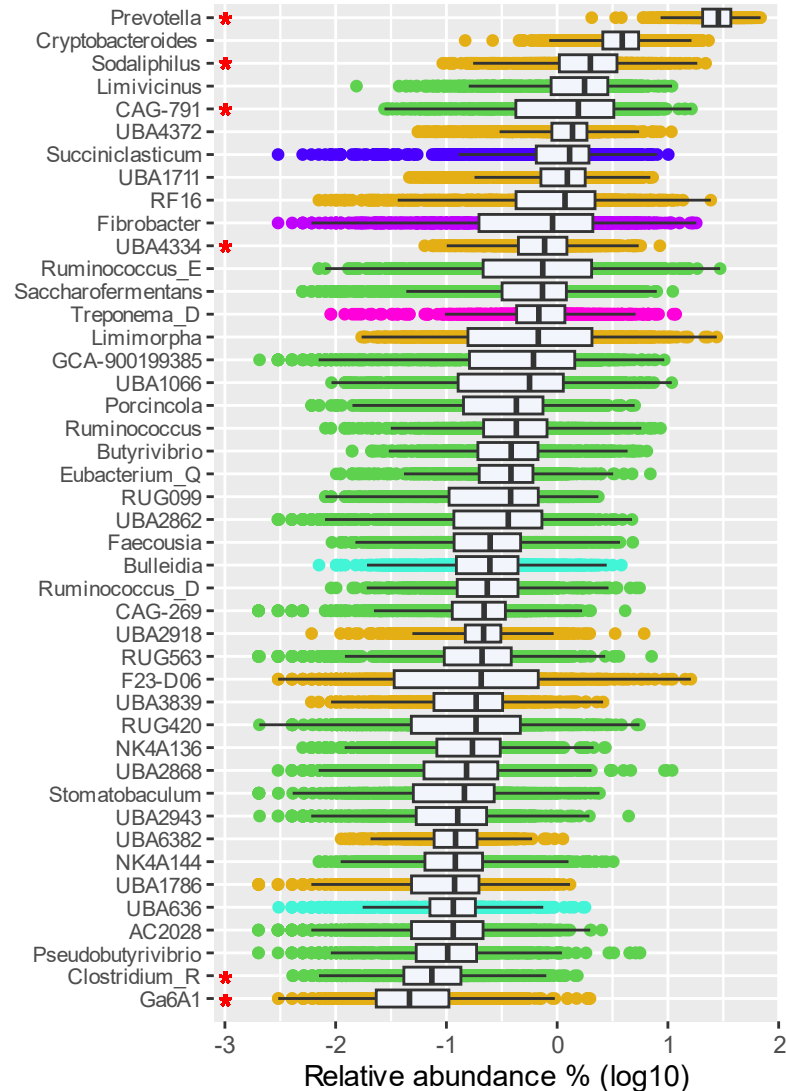

Supplement: Supplementary file 4 — Supplementary Material 3. [file 40168_2024_1791_MOESM3_ESM.pdf]

a

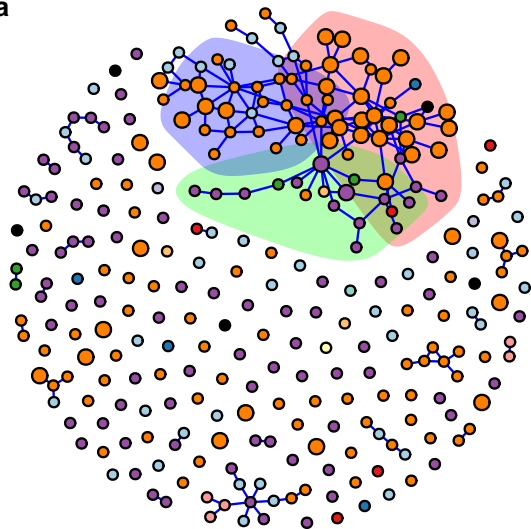

b

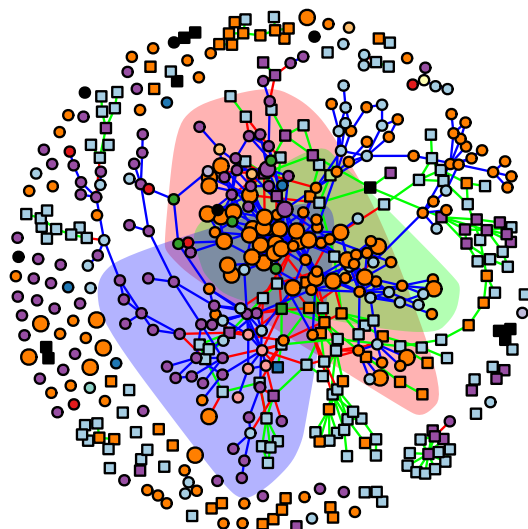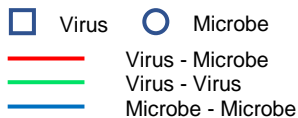

Phylum (microbe / predicted phage host)

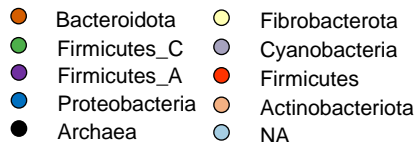

Supplement: Supplementary file 7 — Supplementary Material 6. [file 40168_2024_1791_MOESM6_ESM.pdf]

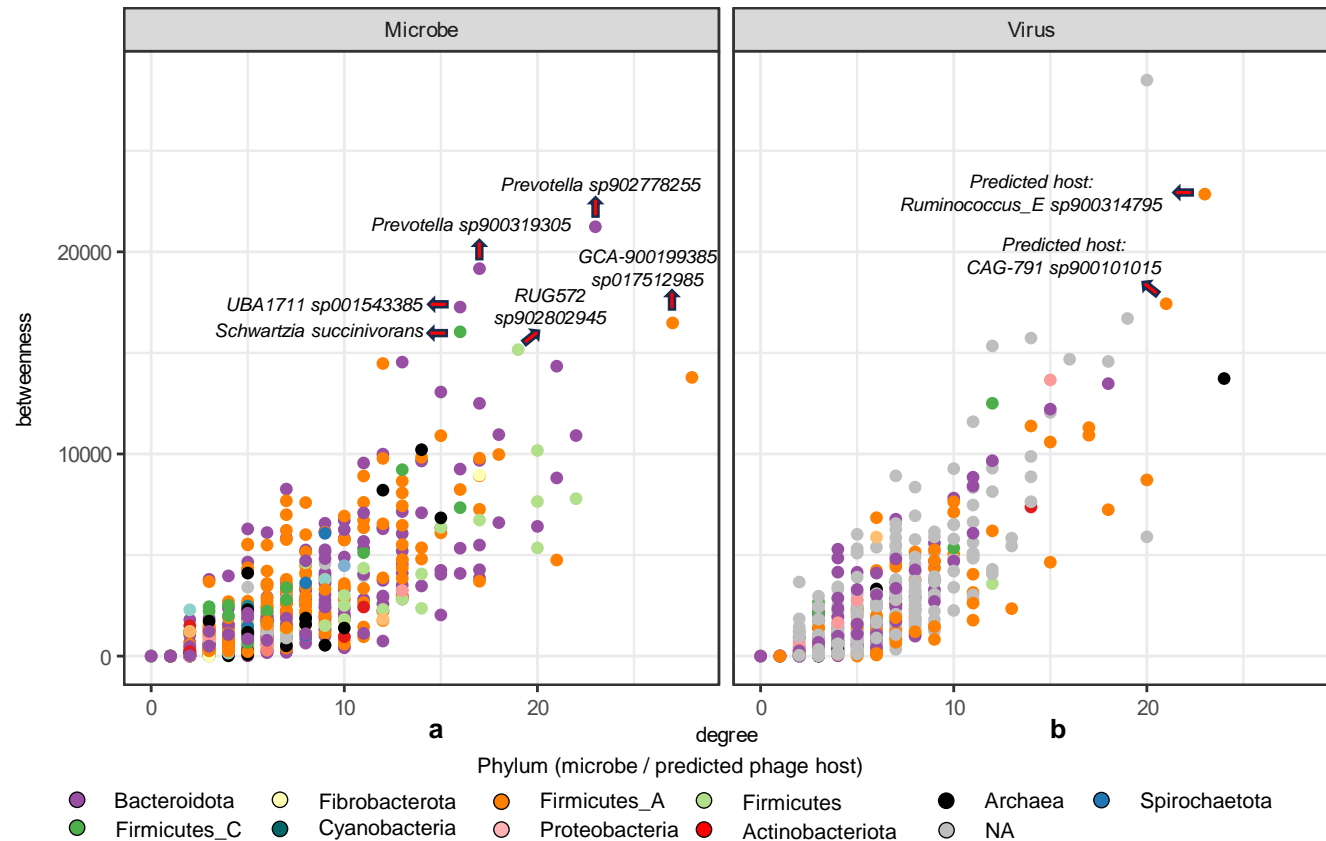

Supplement: Supplementary file 8 — Supplementary Material 7. [file 40168_2024_1791_MOESM7_ESM.pdf]

# Dietary composition

**a**

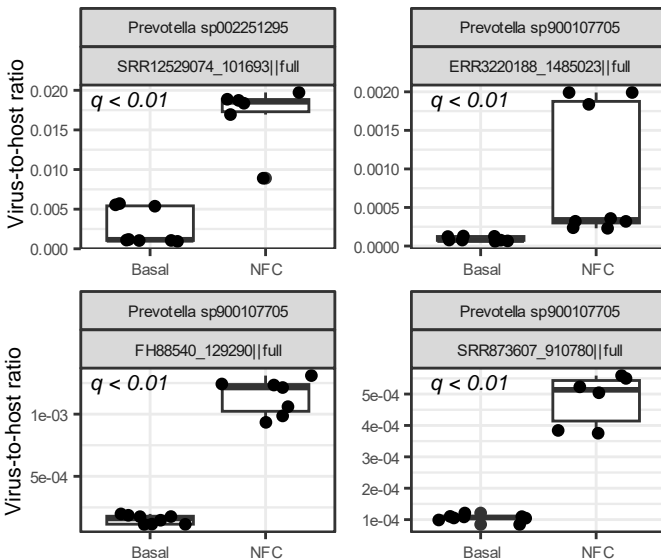

**b**

# Methane emission

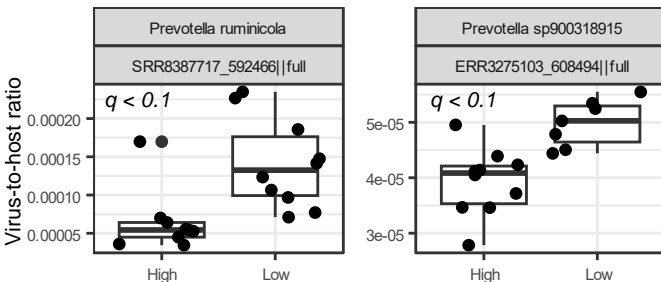

Supplement: Supplementary file 10 — Supplementary Material 9. [file 40168_2024_1791_MOESM9_ESM.pdf]
