# Supplementary material for: Viruses contribute to microbial diversification in the rumen ecosystem and are associated with certain animal production traits
Source: Microbiome. 2024 May 9;12:82. doi: 10.1186/s40168-024-01791-3 (PMC11080232; doi:10.1186/s40168-024-01791-3)
Supplement: Supplementary file 5 — Supplementary Material 4. [file 40168_2024_1791_MOESM4_ESM.pdf]

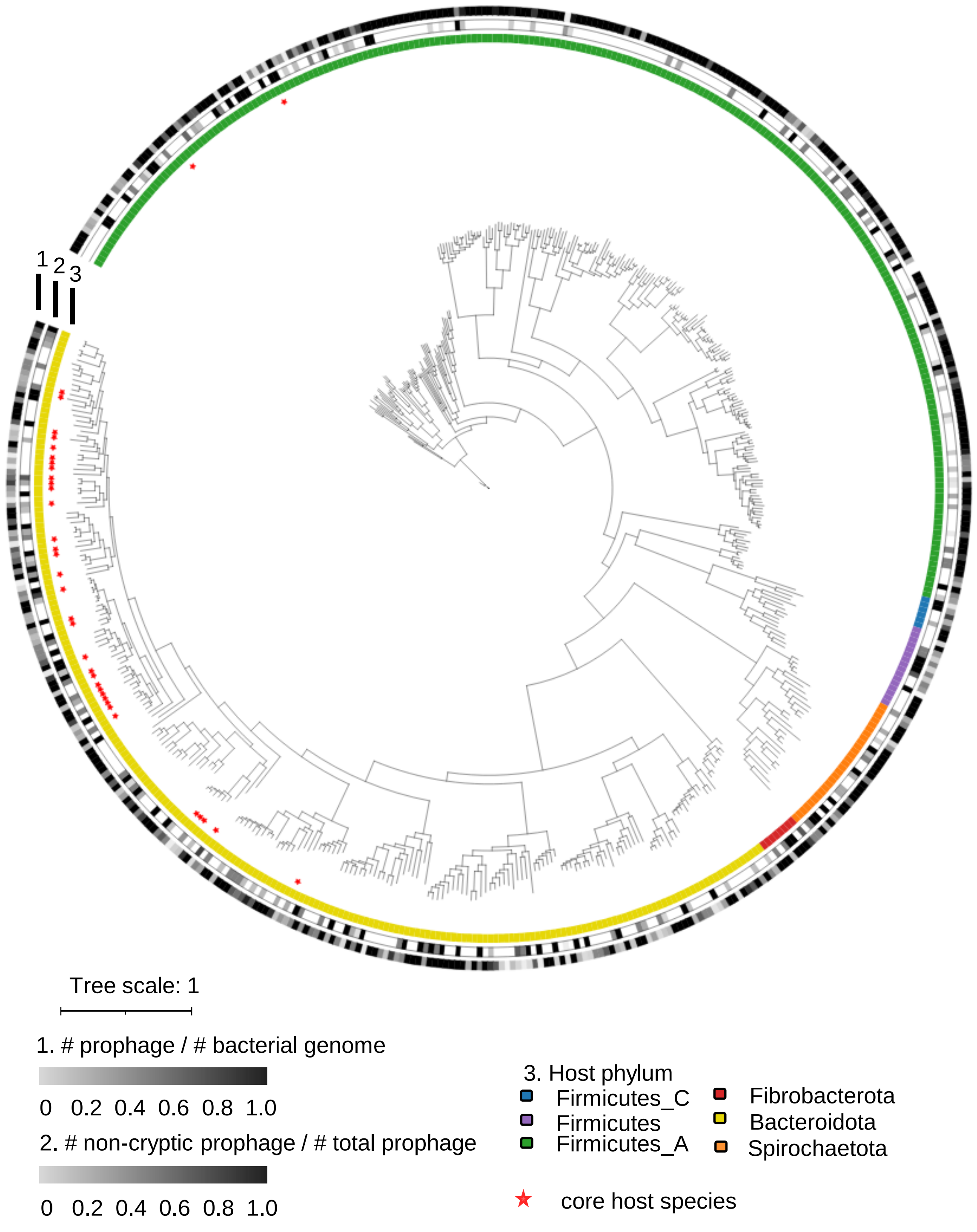
1
2
3
Tree scale: 1
1. # prophage / # bacterial genome
0 0.2 0.4 0.6 0.8 1.0
2. # non-cryptic prophage / # total prophage
0 0.2 0.4 0.6 0.8 1.0
3. Host phylum
Firmicutes_C
Firmicutes
Firmicutes_A
Fibrobacterota
Bacteroidota
Spirochaetota
core host species
