# Supplementary material for: Viruses contribute to microbial diversification in the rumen ecosystem and are associated with certain animal production traits
Source: Microbiome. 2024 May 9;12:82. doi: 10.1186/s40168-024-01791-3 (PMC11080232; doi:10.1186/s40168-024-01791-3)
Supplement: Supplementary file 6 — Supplementary Material 5. [file 40168_2024_1791_MOESM5_ESM.pdf]

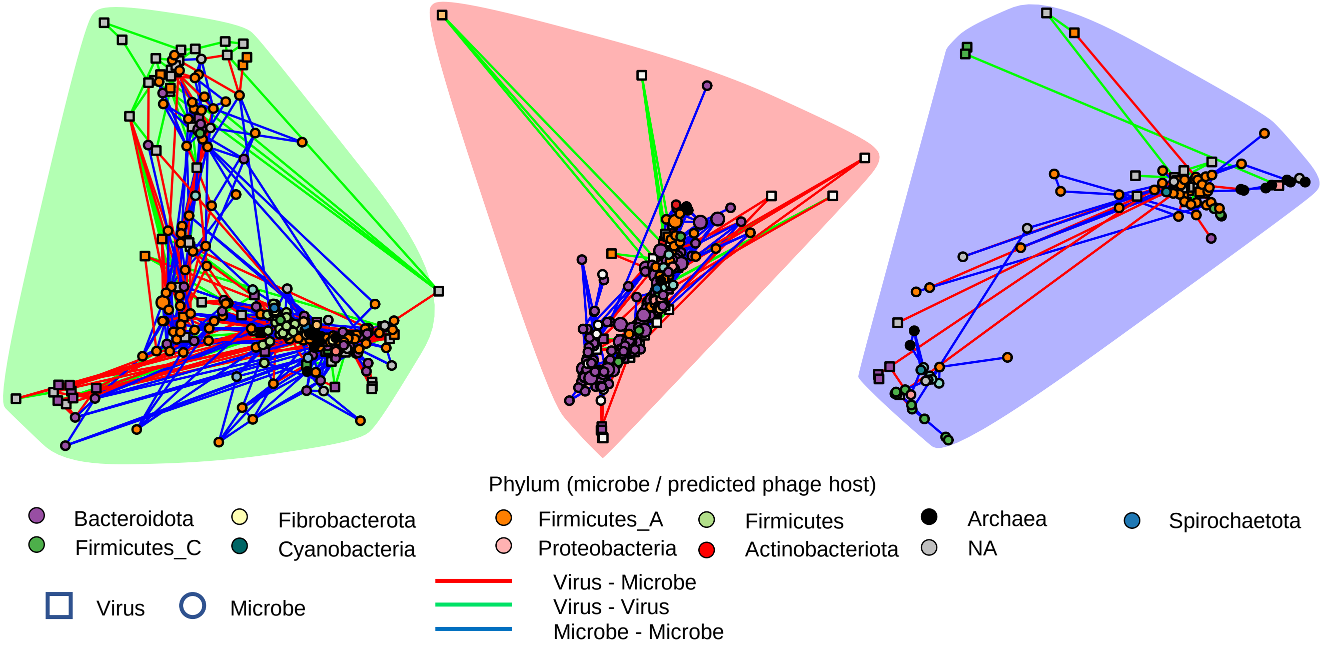

Phylum (microbe / predicted phage host)
Bacteroidota
Firmicutes_C
Fibrobacterota
Cyanobacteria
Firmicutes_A
Proteobacteria
Firmicutes
Actinobacteriota
Archaea
NA
Spirochaetota
Virus
Microbe
Virus - Microbe
Virus - Virus
Microbe - Microbe
