# Supplementary material for: Viruses contribute to microbial diversification in the rumen ecosystem and are associated with certain animal production traits
Source: Microbiome. 2024 May 9;12:82. doi: 10.1186/s40168-024-01791-3 (PMC11080232; doi:10.1186/s40168-024-01791-3)
Supplement: Supplementary file 9 — Supplementary Material 8. [file 40168_2024_1791_MOESM8_ESM.pdf]

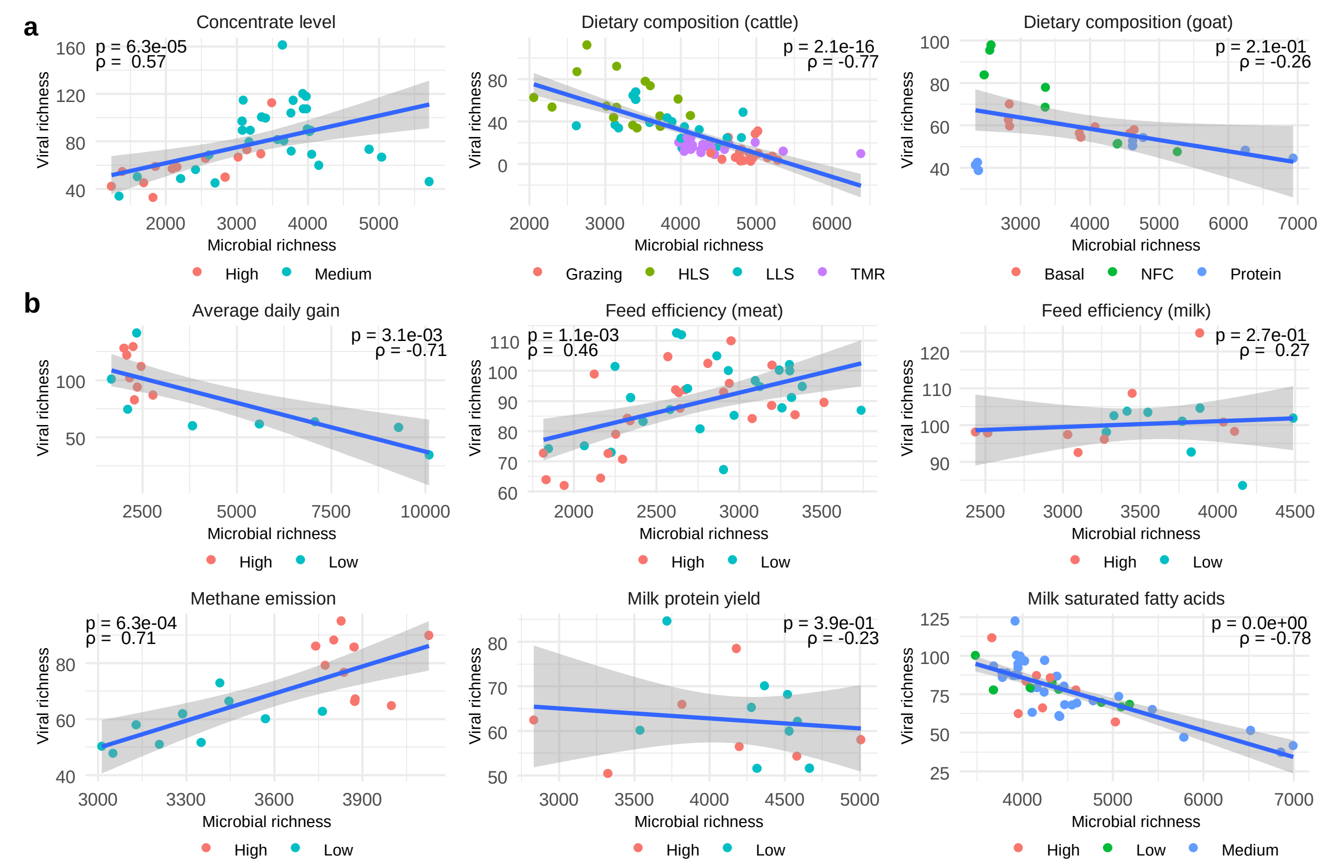

a
Concentrate level
p = 6.3e-05
ρ = 0.57
High
Medium
Dietary composition (cattle)
p = 2.1e-16
ρ = -0.77
Grazing
HLS
LLS
TMR
Dietary composition (goat)
p = 2.1e-01
ρ = -0.26
Basal
NFC
Protein
b
Average daily gain
p = 3.1e-03
ρ = -0.71
High
Low
Feed efficiency (meat)
p = 1.1e-03
ρ = 0.46
High
Low
Feed efficiency (milk)
p = 2.7e-01
ρ = 0.27
High
Low
Methane emission
p = 6.3e-04
ρ = 0.71
High
Low
Milk protein yield
p = 3.9e-01
ρ = -0.23
High
Low
Milk saturated fatty acids
p = 0.0e+00
ρ = -0.78
High
Low
Medium
Viral richness
Microbial richness
